# Cancer metabolism

**DOI:** 10.1186/s12967-021-02753-1

**Published:** 2021-02-25

**Authors:** Violena Pietrobon

**Affiliations:** Refuge Biotechnologies, Menlo Park, CA United States

We are delighted to announce the launch of a new section in the *Journal of Translational Medicine*, ‘Cancer metabolism’. Tumor cells reprogram nutrient acquisition and metabolic pathways to support abnormal growth and metastasis, a dysfunctional process that is a hallmark of cancer. This new section will offer a solid framework to promote and disseminate innovative studies examining metabolic reprogramming in cancer cells, including fatty acid, mitochondrial, glycolytic and other metabolic networks.

We are interested in research that investigates and characterizes metabolic alterations in tumors at the molecular level by explaining why this reprogramming occurs and identifying links between such phenomena and cell proliferation and metastasis. We will also consider studies regarding the correlation between genomic mutations, gene expression, epigenetic regulation and metabolic pathway alterations. Of particular interest are investigations focused on translational and clinical aspects and those regarding metabolically targeted pharmacological therapies, with emphasis on patient responsiveness.

One of the hallmarks of cancer cells is their ability to reprogram cellular metabolism to comply with their biosynthetic, bioenergetic and redox demands, thus providing a selective growth advantage and sustained proliferative capacity. Metabolic reprogramming is largely a consequence of oncogenic mutations and can be defined as a multitude of pathways that are enhanced or suppressed in cancer cells compared to normal tissues. Such alterations are pivotal to acquire and transform nutrients from the tumor microenvironment, which is often nutrient-depleted. Tumorigenesis-associated metabolic alterations affect various stages of the metabolism circuit, such as increasing metabolite influx, modifying nutrient processing and shaping the tumor microenvironment itself. Overall, biosynthetic networks are a crucial feature in cancer metabolism as they enable tumor cells to synthesize the macromolecules required to sustain proliferation and thus, tumor growth.

Many of the aberrantly activated oncogenes that are commonly observed in different types of cancer function in the PI3K-AKT-mTOR pathway. Moreover, MYC gain-of-function is associated with many cancers and leads to the upregulation of genes involved in anabolic growth and nucleotide biosynthesis. Alternatively, p53 is an important regulator of metabolism and oxidative stress, in addition to its role as a tumor suppressor. It has been demonstrated that loss of p53 increases glycolytic flux, promoting the anabolism and redox balance. Indeed, redox homeostasis is essential during both tumorigenesis and metastasis, as the increase in metabolic activity augments the levels of reactive oxygen species (ROS). ROS-dependent signaling leads to further activation of pathways that promote cell proliferation and metabolic adaptation. However, high levels of ROS can also cause cellular damage and require a well-orchestrated machinery to offset their negative effects.

Aerobic glycolysis is one of the most well-studied examples of a metabolic pathway that is reprogrammed in cancer cells. Glucose is the most abundant nutrient in the bloodstream, and it is catabolized to pyruvate through oxidative glycolysis in normal tissues, with the final aim of producing energy in the form of ATP. Between 1924 and 1927, Otto Warburg described how cancer cellular metabolism shifts from oxidative to glycolytic even in the presence of physiological levels of oxygen. As a result, pyruvate is converted into lactate and secreted into the extracellular microenvironment. Tumors are often characterized by a significant increase in glucose consumption due to genomic mutations (oncogene activation or tumor suppressor loss-of-function) or hypoxic areas. Hypoxia is often associated with poor prognosis in patients and dramatically reshapes the transcriptional landscape, which profoundly impacts metabolic networks. Hypoxia or anoxia are caused by lack of an appropriate vascular system in the tumor core, which also implies reduced nutrient accessibility for cancer cells.

Therefore, alternative adaptive forms of metabolism are essential to ensure the survival and proliferation of malignant cells in these conditions, including oxidation of fatty acids, autophagy and micropinocytosis. Autophagy relies on the recycling of intracellular biomass and, despite promoting cell survival during periods of starvation, it is incapable of supporting tumor growth. Fatty acid synthesis requires acetyl-CoA, and in vitro studies have demonstrated that glucose is an important source of acetyl-CoA. However, glutamine and acetate can offer alternative carbon sources when cells experience hypoxic conditions. Glutamine is a source of nitrogen and carbon, and is the most abundant circulating amino acid in humans. For example, actively dividing cells synthesize nucleotides *de novo*, non-essential amino acids and polyamines which are nitrogen-containing molecules. After glucose and glutamine, serine is the third most consumed metabolite by cancer cells. Serine is the main contributor of one-carbon units in malignant cells, which are used in the biosynthesis of thymidine monophosphate and purines. Moreover, part of the serine pool is also converted into glycine. One-carbon molecules are also required for methylation processes, which affect epigenetic regulation and gene expression. Finally, another crucial amino acid is arginine, which is imported into cells and converted into ornithine, a precursor of polyamine synthesis.

The study of cancer cell metabolism is important to provide new insights into possible therapeutic targets and strategies. Metabolic reprogramming profoundly affects the features of the cells and of the tumor microenvironment, creating hostile conditions for T cell proliferation and survival and negatively affecting the host immune response. The objective of the *Journal of Translational Medicine* is to offer a connection between basic and clinical science, supporting scientific discussion and diffusion of innovative concepts through open access and a high standard peer-review process. The “Cancer metabolism” section will ensure the publication of high quality and internationally competitive studies. The Editorial Board is looking forward to receiving your manuscripts.

